# Risk factors for mortality in patients with *Pseudomonas aeruginosa* bacteremia; retrospective study of impact of combination antimicrobial therapy

**DOI:** 10.1186/1471-2334-14-161

**Published:** 2014-03-24

**Authors:** Youn Jeong Kim, Yoon Hee Jun, Yang Ree Kim, Kang Gyun Park, Yeon Joon Park, Ji Young Kang, Sang Il Kim

**Affiliations:** 1 Division of infectious disease, Department of Internal Medicine, Seoul St. Mary's hospital, College of Medicine, The Catholic University of Korea, Seoul, Korea; 2 Department of Laboratory Medicine,Seoul St. Mary's hospital, College of Medicine, The Catholic University of Korea, Seoul, Korea; 3 Division of Pulmonology, Department of Internal Medicine, Seoul St. Mary's hospital, College of Medicine, The Catholic University of Korea, 222 Banpo-daero, Seocho-gu, Seoul 137-701, Republic of Korea

**Keywords:** Pseudomonas aeruginosa, Bacteremia, Risk factors, Anti-bacterial agents, Combination, Mortality

## Abstract

**Background:**

Whether the combination of antimicrobial therapy is a factor in mortality in *Pseudomonas aeruginosa* bacteremia remains to be elucidated. This study investigated the risk factors for mortality in *P. aeruginosa* bacteremia patients and the influence of adequate antimicrobial therapy and combination therapy on clinical outcomes.

**Methods:**

This retrospective study analyzed data of 234 patients with *P. aeruginosa* bacteremia at a 1,200-bed tertiary teaching university hospital in South Korea between January 2010 and December 2012. Factors associated with mortality were determined. Mortality was compared in patients with adequate empirical and targeted combination therapy, and monotherapy, and inappropriate therapy.

**Results:**

A total of 141 (60.3%) patients were given appropriate empirical antibiotic treatment (combination therapy in 38 and monotherapy in 103). Among 183 patients (78.2%) who finally received appropriate targeted treatment, 42 had combination therapy and 141 had monotherapy. The percentage of patients receiving empirical combination therapy was slightly, but not significantly higher, in the survivor group than in the nonsurvivor group (17.0% [31/182] vs. 13.5% [7/52], p = 0.74). A similar tendency was demonstrated for targeted combination therapy (19.8% [36/182] vs. 11.5% [6/52], respectively; p = 0.31). However, in a subgroup analysis of data from patients (n = 54) with an absolute neutrophil count less than 500/mm^3^, the patients who had appropriate empirical or targeted combination therapy showed better outcomes than those who underwent monotherapy or inappropriate therapy (p < 0.05). Mechanical ventilation (odds ratio [OR], 6.93; 95% confidence interval [CI], 2.64–18.11; p = 0.0001), the use of a central venous catheter (OR, 2.95; 95% CI, 1.35–6.43; p = 0.007), a high Acute Physiology and Chronic Health Evaluation II score (OR, 4.65; 95% CI, 1.95–11.04; p = 0.0001), and presence of septic shock (OR, 2.91; 95% CI, 1.33–6.38; p = 0.007) were independent risk factors for 14-day mortality.

**Conclusions:**

Disease severity was a critical factor for mortality in our patients with *P. aeruginosa* bacteremia. Overall, combination therapy had no significant effect on 14-day mortality compared with monotherapy. However, appropriate combination therapy showed a favorable effect on survival in patients with febrile neutropenia.

## Background

*Pseudomonas aeruginosa* represents a common cause of nosocomial infection. Immunocompromised patients such as those with malignancy or neutropenia are at high risk of bacteremia, and *P.aeruginosa* is one of the commonly isolated pathogens associated with bacteremia in such patients [1,2]. Despite advances in antimicrobial therapy, *P.aeruginosa* infection remains associated with high mortality ranging of 18% - 61% [3].

The therapeutic options for *P. aeruginosa* infection are limited owing to the intrinsic resistance of the bacterium to commonly used antibiotics and the increase in multidrug resistance. The use of more than one kind of antibiotic has been known to be effective for certain patients; the use of a combination of at least two drugs was demonstrated to have a synergistic or additive effect in lowering the risk of receiving an inappropriate empirical therapy, and to prevent the emergence of resistant organisms [4]. Some studies reported that a combination therapy in patients with gram-negative bacteremia resulted in better outcomes than monotherapy [5,6]. However, the effects of combination therapies for *P. aeruginosa* infection remain unclear.

The risk factors for mortality in patients with bacteremia are reported to be severe sepsis, neutropenia, and multidrug resistance [7-10]. Whether the adequacy of antimicrobial therapy is a factor for mortality in *P. aeruginosa* bacteremia remains to be elucidated [7,11-13]. In this study, we identified the risk factors for mortality and investigated the effect of the adequacy of antimicrobial therapy in patients with *P. aeruginosa* bacteremia. We also analyzed and compared the effects of combination therapy and monotherapy on 14-day mortality.

## Methods

### Study design

A retrospective study was performed on data from patients (>18 years old) with confirmed clinical signs of *P. aeruginosa* infection between January 2010 and December 2012 at a 1200-bed tertiary teaching hospital in South Korea. *P. aeruginosa* was isolated from at least one set of blood cultures of samples collected from the patients. Only the first bacteremia episode in each patient was included in this study. We assessed the severity of underlying disease using the Acute Physiology and Chronic Health Evaluation (APACHE) II scoring system and the Charlson comorbidity index. We used 14-day overall mortality as the main outcome for the assessment of mortality in patients.

Empirical antimicrobial therapy was defined according to the initial antimicrobial therapy regimens that were administered within 24 hours after blood culture samples were obtained, and before results of susceptibility tests were known. Targeted antimicrobial therapy was defined as specific antibiotics given within 24 hours after the results of antimicrobial susceptibility. Antimicrobial therapy was considered appropriate when the strain showed *in vitro* susceptibility to the antibiotics administered, and the dosages of the drugs were adequate according to current guidelines [14]. An appropriate combination therapy was defined if two or more antibiotics showed *in vitro* susceptibility. Appropriate monotherapy was defined as treatment with only one active antibiotic. Aminoglycoside monotherapy was also defined as inadequate therapy. Neutropenia was defined as an absolute neutrophil count (ANC) < 500/mm^3^ at the time of bacteremia. Multidrug resistance was defined when the strain was resistant to three or more antipseudomonal anti-microbial categories (ciprofloxacin, ceftazidime, piperacillin/tazobactam, meropenem, and amikacin) [15,16]. Infection was assessed according to the criteria established by the Centers for Disease Control and Prevention, and patients were considered to have contracted the infection when *P. aeruginosa* was isolated from a sterile site in patients with definite clinical signs of infection [17]. Septic shock was defined in the published guidelines [18].

### Microbiological examination

Identification of *P. aeruginosa* in blood samples was performed using a VITEK 2 automated system (bioMérieux, Marcy l’Etoile, France). Susceptibility results were interpreted according to the guidelines established by the Clinical and Laboratory Standards Institute [19]. Carbapenem resistance was defined as nonsusceptibility to meropenem and/or imipenem *in vitro*, and isolates with intermediate resistance were regarded as resistant.

### Statistically analysis

The Student *t*-test or the Mann–Whitney *U* test were used for analysis of continuous variables, and the χ^2^ test or Fisher’s exact test were used for categorical variables. Multivariate analysis using multiple logistic regression was performed for statistically significant factors in the univariate analysis to determine the risk factors associated with 14-day overall mortality. Statistical analysis was performed using SPSS 13.0 (SPSS Inc., Chicago, IL, USA), and p < 0.05 was considered statistically significant.

### Ethical statement

This study was approved by the institutional review board of Seoul St. Mary’s Hospital, Seoul (KC14RISI0033).

## Results

### Demographic characteristics

During the study period, a total of 234 patients (92 male, 142 female) with *P.aeruginosa* bacteremia were included. The most common underlying disease was malignancy (n = 68, 29.1%), followed by hypertension (n = 52, 22.2%) and diabetes mellitus (n = 47, 20.1%). *P. aeruginosa* resistance to carbapenem was found in 50.4% (n = 118) of patients. The presumed sources of bacteremia were the respiratory tract (n = 70, 29.9%), abdominal cavity (n = 42, 17.9%), urinary tract (n = 18, 7.7%), postoperative wound (n = 11, 4.7%), vascular catheter (n = 4, 1.7%), and other unknown sources (n = 89, 38%). The overall 14-day mortality was 22.2% (n = 52), and almost half (n = 25) died within 24 hours after the onset of bacteremia.

Table 1 shows the comparison of the demographic characteristics of the survivors and nonsurvivors. Neutropenia was more frequent among the patients who died than among those who survived (34.6% [18/52] vs. 19.8% [36/182], p = 0.02). Nonsurvivors showed a higher APACHE II score (18 vs. 11, p = 0.0001) and had a higher rate of septic shock (53.8% [28/52] vs. 18.7% [34/182], p = 0.0001) than the survivors. The use of a central venous catheter (69.2% [36/52] vs. 33.5% [61/182], p = 0.0001), foley catheter (53.8% [28/52] vs. 12.1% [22/182], p = 0.0001) and mechanical ventilator (38.5% [20/52] vs. 6.6%[12/182], p = 0.0001) was significantly more frequent among the nonsurvivors than among the survivors.

**Table 1:** **Comparison of demographic characteristics of survivors and non survivors of** ***Pseudomonas aeruginosa*** **bacteremia**

	**Total (n = 234)**	**Non survivors (n = 52)**	**Survivors (n = 182)**	***P *** **value**
Age, median years (range)	57 (19 ~ 93)	60.5 (19 ~ 84)	56 (21 ~ 93)	0.35
Male sex	92 (39.3%)	23 (44.2%)	69 (37.9%)	0.41
Underlying disease				
Diabetes mellitus	47 (20.1%)	8 (15.4%)	39 (21.4%)	0.34
Hypertension	52 (22.2%)	11 (21.2%)	41 (22.5%)	0.83
Liver cirrhosis	13 (5.6%)	2 (3.8%)	11 (6.0%)	0.54
Transplant	34 (14.5%)	10 (19.2%)	24 (13.2%)	0.27
Solid organ	8 (3.4%)	1 (1.9%)	7 (3.8%)	
Bone marrow	26 (11.1%)	9 (17.3%)	17 (9.3%)	
Malignancy	68 (29.1%)	14 (26.9%)	54 (29.7%)	0.70
Dialysis	11 (4.7%)	2 (3.8%)	9 (4.9%)	0.74
Neutropenia	54 (23.1%)	18 (34.6%)	36 (19.8%)	0.02
Hospitalization in the preceding 90 days	98 (41.9%)	26 (50.0%)	72 (39.6%)	0.18
APACHE II score, median (IQR)	12 (0 ~ 40)	18 (5 ~ 40)	11 (0 ~ 28)	0.0001
Charlson comobidity index, median (IQR)	3 (0 ~ 13)	4 (1 ~ 10)	4 (0 ~ 13)	0.57
Septic shock	62 (26.7%)	28 (53.8%)	34 (18.7%)	0.0001
Invasive procedure				
Central venous catheter	97 (41.5%)	36 (69.2%)	61 (33.5%)	0.0001
Surgical drainage	39 (16.7%)	8 (15.4%)	31 (17.0%)	0.78
Foley catheter	50 (21.4%)	28 (53.8%)	22 (12.1%)	0.0001
Mechanical ventilator	32 (13.7%)	20 (38.5%)	12 (6.6%)	0.0001
The length of stay before bacteremia, median days (IQR)	1 (0 ~ 92)	1 (0 ~ 28)	2.5 (0 ~ 92)	0.2
Source of bacteremia				0.006
Pneumonia	70 (29.9%)	27 (51.9%)	43 (23.6%)	
Urinary tract	18 (7.7%)	2 (3.8%)	16 (8.8%)	
Vascular catheter-related	4 (1.7%)	1 (1.9%)	3 (1.6%)	
Intra-abdomen	42 (17.9%)	7 (13.5%)	35 (19.2%)	
Postoperative wound	11 (4.7%)	1 (1.9%)	10 (5.5%)	
Unknown	89 (38.0%)	14 (26.9%)	75 (41.2%)	
Length of hospital stay, median days (IQR)	17 (1 ~ 364)	12 (1 ~ 364)	18 (1 ~ 92)	0.08
Carbapenem resistance	118 (50.4%)	26 (50.0%)	92 (50.5%)	0.94
Multidrug resistance	6 (2.6%)	3 (5.8%)	3 (1.6%)	0.09

In the univariate analysis, 14-day mortality was associated with neutropenia (odds ratio [OR], 2.14; 95% confidence interval [CI], 1.09–4.22; p = 0.03), the use of a mechanical ventilator (OR, 8.85; 95% CI, 3.94–19.88; p = 0.0001), the use of a central venous catheter (OR, 4.46; 95% CI, 2.29–8.67; p = 0.0001), a high APACHE II score (OR, 8.88; 95% CI, 4.06–19.38; p = 0.0001), and the presence of septic shock (OR, 5.08; 95% CI, 2.63–9.83; p = 0.0001) (Table 2). The multivariate analysis revealed that the use of a mechanical ventilator (OR, 6.93; 95% CI, 2.64–18.11; p = 0.0001), the use of a central venous catheter (OR, 2.95; 95% CI, 1.35–6.43; p = 0.007), a high APACHE II score (OR, 4.65; 95% CI, 1.95–11.04; p = 0.0001), and the presence of septic shock (OR, 2.91; 95% CI, 1.33–6.38; p = 0.007) were the independent risk factors for 14-day mortality (Table 2).

**Table 2:** **Risk factors associated with 14**-**day mortality in patients with*P. aeruginosa*bacteremia**

	**Univariate analysis**			**Multivariate analysis**		
**Risk factor**	**OR**	**95% CI**	***P *** **value**	**OR**	**95% CI**	***P *** **value**
Age > 60 (years)	1.42	0.77-2.64	0.26			
Neutropenia	2.14	1.09-4.22	0.03	1.45	0.63-3.33	0.37
Mechanical ventilator	8.85	3.94-19.88	0.0001	6.93	2.64-18.11	0.0001
Central venous catheter	4.46	2.29-8.67	0.0001	2.95	1.35-6.43	0.007
APACHE II score ≥ 13	8.88	4.06-19.38	0.0001	4.65	1.95-11.04	0.0001
Septic shock	5.08	2.63-9.83	0.0001	2.91	1.33-6.38	0.007
Carbapenem resistance	1.02	0.55-1.89	0.94			
Multidrug resistance	3.65	0.72-18.67	0.12			
Pneumonia as a source of bacteremia	1.01	0.51-2.01	0.96			
Empirical therapy						
Appropriate combination therapy	0.82	0.31-2.14	0.69			
Appropriate monotherapy	1.17	0.59-2.28	0.64			
Inappropriate therapy	1.0 (ref)					
Targeted therapy						
Appropriate combination therapy	0.44	0.37-1.61	0.49			
Appropriate monotherapy	0.78	0.15-1.27	0.13			
Inappropriate therapy	1.0 (ref)					

*OR* = odd rations; *CI* = confidential interval; *CR* = carbapenem-resistance.

A total of 141 (60.3%) patients were given appropriate empirical antibiotic treatment, which included combination therapy in 38 patients (16.2%) and monotherapy in 103 patients (44.0%). Among 183 patients (78.2%) who were finally treated with appropriate appropriate targeted treatment, 42 (17.9%) were given combination therapy and 141 (60.3%) were given monotherapy. Table 3 describes the administered adequate antibiotics in detail. Adequate empirical antimicrobials used in monotherapy were as follows: 88 β-lactam, 8 fluoroquinolone, and 7 colistin, whereas targeted monotherapy consisted of 114 β-lactam, 20 fluoroquinolone, and 7 colistin. β-lactam and aminoglycoside was the most frequent combination in patients treated with adequate empirical combination therapy (84.2% [32/38]), and in adequate targeted combination therapy (78.6% [33/42]). The appropriateness of empirical therapy was not significantly different between the survivor and nonsurvivor groups (61.5% [32/52] vs. 60.4% [110/182], p = 0.89), and the appropriateness of targeted therapy was also not different between the two groups (73.1% [38/52] vs. 79.7% [145/182], p = 0.31).

**Table 3:** Description of administered antibiotics in patients receiving adequate treatment

**Adequate monotherapy**		
	**Empirical (n = 103)**	**Targeted (n = 141)**
ß-lactam	88 (85.4%)	114 (80.9%)
Antipseudomonal penicillin	24 (23.3%)	31 (21.9%)
Cephalosporin	45 (43.7%)	36 (25.5%)
Carbapenem	19 (18.4%)	47 (33.3%)
Fluoroquinolones	8 (7.8%)	20 (14.2%)
Colistin	7 (6.8%)	7 (4.9%)
Adequate combination therapy		
	Empirical (n = 38)	Targeted (n = 42)
ß-lactam + aminoglycosides	32 (84.2%)	33 (78.6%)
Antipseudomonal penicillin	6 (68.4%)	25 (59.6%)
Cephalosporin	26 (15.8%)	3 (7.1%)
Carbapenem	0	5 (11.9%)
ß-lactam + fluoroquinolones	5 (13.2%)	7 (16.7%)
Antipseudomonal penicillin	3 (7.9%)	2 (4.8%)
Cephalosporin	2 (5.3%)	4 (9.5%)
Carbapenem	0	1 (2.4%)
Colistin + fluoroquinolones	1 (2.6%)	1 (2.4%)
Colistin + aminoglycoside	0	1 (2.4%)

Patients with *P.aeruginosa* bacteremia who received combination empirical therapy or targeted therapy did not show better outcomes than those who receivied monotherapy or inadequate therapy (Table 4). The percentage of patients receiving empirical combination therapy was slightly, but not significantly higher, in the survivor group than in the nonsurvivor group (17.0% [31/182] vs. 13.5% [7/52], p = 0.74). A similar tendency was found in the group receiving targeted therapy (19.8% [36/182] vs. 11.5% [6/52], respectively; p = 0.31). However, a subgroup analysis of data from patients (n = 54) with an ANC < 500/mm^3^ indicated that that those who underwent appropriate empirical combination therapy showed better outcomes than those who underwent appropriate empirical monotherapy or inappropriate therapy (p = 0.001). In addition, patients who underwent appropriate targeted combination therapy also showed a more favorable outcome than those who underwent appropriate targeted monotherapy or inappropriate therapy (p = 0.01) (Table 4).

**Table 4:** Comparison of outcomes according to adequacy of antibiotics

			**Survivor (n = 182)**	**Non survivor (n = 52)**	**P value**
All patients (n = 234)	Empirical	Combination	31 (17.0%)	7 (13.5%)	0.74
		Monotherapy	78 (42.9%)	25 (48.1%)	
		Inappropriate	31 (17.0%)	7 (13.5%)	
	Targeted	Combination	36 (19.8%)	6 (11.5%)	0.31
		Monotherapy	109 (59.9%)	32 (61.5%)	
		Inappropriate	37 (20.3%)	14 (26.9%)	
Patients with neutropenia (n = 54)	Empirical	Combination	19 (52.7%)	4 (22.2%)	0.001
		Monotherapy	16 (44.4%)	7 (38.8%)	
		Inadequate	1 (2.7%)	7 (38.8%)	
	Targeted	Combination	21 (58.3%)	10 (55.5%)	0.01
		Monotherapy	14 (38.8%)	3 (16.7%)	
		Inadequate	1 (2.7%)	5 (27.7%)	

## Discussion

The effect of appropriate therapies and combination therapies on mortality in patients with *P. aeruginosa* bacteremia remains to be clarified. In this study, the overall in-hospital 14-day mortality rate in the patients with *P. aeruginosa* bacteremia was as high as 22.2%. The independent predictors for mortality among patients with *P. aeruginosa* bacteremia were the use of a mechanical ventilator, the use of a central venous catheter, a high APACHE II score, and a clinical presentation of septic shock. We did not demonstrate a beneficial effect of adequate empirical or targeted combination therapy on survival, although there was a tendency towards a protective effect on mortality. However, in the subgroup with neutropenia, adequate empirical or targeted combination therapy was associated with a significantly lower 14-day mortality rate. The condition of patients with *P. aeruginosa* bacteremia deteriorated rapidly, and most nonsurvivors died on the day of onset. Therefore, early appropriate treatment plays an important role in the outcome among *P. aeruginosa* bacteremia patients, and some studies have reported that inappropriate antibiotic therapy is associated with poor prognosis [11,20]. There are several reports that a clinical presentation of sepsis is a strong predictor of mortality consistent with the present study [7,13,21]. As neutropenia may be a factor for increased mortality in patients with hematologic or solid malignancy, we analyzed the relationship between adequacy of antibiotic therapy and mortality after stratifying the cases in our study according to the presence of neutropenia [10,22]. Our results demonstrated that adequate combination antimicrobial therapy was associated with a decreased mortality rate in patients with neutropenia. Combination therapy is advantageous in terms of preventing the emergence of resistant organisms, its synergistic effect, and its ability to increase the likelihood of organisms being susceptible to at least one of the component antibiotics [4]. A meta-analysis by Safdar et al. demonstrated that combination therapy could reduce the mortality rate in patients with *P. aeruginosa* infection; however, another study indicated that combination therapy did not affect patient outcomes [21,23]. There is controversy whether combination therapy for *P. aeruginosa* is superior to monotherapy in improving the survival of the patients with *P. aeruginosa* bacteremia. Our study showed that combination therapy may have a significant beneficial effect in a subgroup of patients with neutropenia One meta-analysis showed that there was no significant difference between ß-lactam-aminoglycoside combination therapy and monotherapy in patients with febrile neutropenia, however the analysis did not target only patients with *P. aeruginosa* infection, and did not investigate the adequacy of antimicrobial therapy [24]. Our results are consistent with those of a prospective observational study that reported that combination therapy was beneficial only in neutropenic patients, although the study included patients with Gram-negative bacteremia, and not specifically *P. aeruginosa* infection [25]. We suggest that combination empirical therapy should be considered for febrile neutropenic patients, if there is a risk of *Pseudomonas* infection. In our non-neutropenic patients, combination therapy for *P. aeruginosa* bacteremia did not appear to confer a significant additional beneficial effect, and disease severity such as the presence of septic shock and high APACHE II score played an important role in the treatment outcome.

In our study, approximately 50% of all patients were infected with carbapenem-resistant *P. aeruginosa*, indicating a high prevalence of antibiotic resistance in our hospital compared with the approximately 30% prevalence rate reported for the general Korean population [26]. Recently, cases of carbapenem resistance of gram-negative isolates have been increasing, as in our hospital [26]. The high prevalence of cases is likely the result of our hospital population, including a high percentage of malignancy cases and transplant recipients. Carbapenem resistance or multidrug resistance was not an independent factor for higher 14-day mortality in our study. There is some controversy with regard to the effect of antibiotic resistance on mortality. Some studies have reported that the clinical presentation or the use of appropriate antibiotics, rather than resistance, were predictors of mortality in patients with *P. aeruginosa* bacteremia [27,28]. Further studies are required to clarify this point.

The major limitation of this study was its retrospective nature in a single center and diversity of underlying disease and condition of bacteremia. Second, the choice of antibiotics depended on the physician’s opinion, which could be a source of bias. We did not analyze outcome based on the antibiotic administered. Third, appropriate therapy was defined as the administration of antibiotics to which the isolate had *in vitro* susceptibility. In our study, colistin monotherapy was defined as adequate if *P. aeruginosa* was susceptible to colistin *in vitro*. Colistin has been reintroduced for the treatment of multidrug resistant Gram-negative bacilli infections, and there have been reports of the synergy of colistin combined with other antibiotics [29]. However, it remains questionable in clinical practice whether colistin monotherapy is inferior to combination therapy in patients with *P. aeruginosa* infection, especially multidrug-resistant species.

## Conclusion

The mortality rate of *P. aeruginosa* bacteremia remains high, despite advances in antibiotic therapy. High APACHE II score and presence of septic shock was the critical factor for mortality in *P. aeruginosa* bacteremia, and combination therapy did not significantly reduce the overall 14-day mortality rate. However, in cases with febrile neutropenia, combination therapy showed a beneficial effect on survival, and early appropriate combination treatment was associated with a positive effect on patient outcomes.

## Competing interests

The authors declare that they have no competing interests.

## Authors’ contributions

YJK participated in the design of the study, performed the statistical analysis, wrote the paper and drafted the manuscript. YHJ participated in its design and performed critical review. YRK and YJP performed critical review. KGP collected the data. JYK and SIK participated in its design and coordination, and performed critical review. All authors read and approved the final manuscript.

## Pre-publication history

The pre-publication history for this paper can be accessed here:

http://www.biomedcentral.com/1471-2334/14/161/prepub
